# Flexor tenotomy for mallet toe with penetration of the middle phalanx head by dual-component intramedullary implant following proximal interphalangeal arthrodesis

**DOI:** 10.1016/j.ijscr.2021.106703

**Published:** 2022-01-10

**Authors:** Ichiro Tonogai

**Affiliations:** Department of Orthopedics, Institute of Biomedical Science, Tokushima University Graduate School, 3-18-15 Kuramoto, Tokushima 770-8503, Japan

**Keywords:** Hammer toe, Dual-component intramedullary implant, Arthrodesis, Mallet toe, Flexor tenotomy

## Abstract

**Introduction:**

There is a risk of mallet toe following proximal interphalangeal (PIP) joint fusion for hammertoe. Here we describe a rare case of penetration of the dorsal aspect of the middle phalanx head by the distal portion of a dual-component intramedullary implant during progression of mallet toe that was treated with flexor tenotomy.

**Presentation of case:**

A 59-year-old man underwent uneventful arthrodesis of the third PIP using a dual-component intramedullary implant and presented 6 months later with progressive mallet toe and swelling, pain, and ulceration over the distal interphalangeal joint of the third toe. Imaging showed that the distal portion of the implant had penetrated the dorsal aspect of the middle phalanx head. A longitudinal incision was made over the dorsum of the middle and proximal phalanges of the third toe and the implant was removed. A plantar incision was made at the metatarsophalangeal joint and the flexor tendon was cut to correct the mallet toe deformity. One year later, correction was satisfactory with an acceptable functional outcome and good pain relief.

**Discussion:**

We successfully treated a man with penetration of the dorsal border of the middle phalanx head in the third toe by the distal portion of a dual-component intramedullary implant as a result of mallet toe that developed following PIP arthrodesis, by removing the implant and performing flexor tenotomy.

**Conclusion:**

Addition of flexor tenotomy should be considered when performing PIP arthrodesis in a patient with risk factors for severe mallet toe.

## Highlights

• Dual component intramedullary implant is useful for hammer toe.
• Mallet toe occur after proximal interphalangeal arthrodesis.
• Middle phalangeal head penetration by implant occur
• Flexor tenotomy is useful for mallet toe.

## 1 Introduction

Hammer toe and claw toe are characterized by plantar flexion deformity in the proximal interphalangeal (PIP) joint, and the discrimination between them is based on whether the deformity occurs in the metatarsophalangeal (MTP) joint. Hammer toe is a foot deformity that can be caused by diabetes [1], trauma, imbalance of the intrinsic and extrinsic musculature, a neuromuscular, neurologic, or inflammatory disorder, ill-fitting footwear, neuropathy, peripheral vascular disease, hallux valgus, or congenital etiology. During weight-bearing or gait, insensate and deformed toes are subject to increased pressure and shear stresses, which can result in callus formation, tissue trauma, and ulceration [2]. Lesser toe deformities are a forefoot problem that greatly hinders quality of life [3]. Arthrodesis of a proximal interphalangeal (PIP) joint using a one-piece (single-component) or two-piece (dual-component) intramedullary implant is now the standard treatment for rigid toe deformity or a structural toe deformity not amenable to manual correction.

The one-piece implant (PRO-TOE™; Wright Medical Japan KK, Tokyo, Japan) available in Japan has the disadvantage of requiring more extensive bone resection, which can result in instability of the joint and tendon dysfunction. The more recent two-piece intramedullary implant (Nextra®; Zimmer Biomet, Warsaw, IN, USA) has been available in Japan since 2018. This implant includes an interlocking titanium screw that allows intramedullary PIP arthrodesis for rigid hammer toe. Fazal et al., Ellington et al. and Jay RM et al. have reported good clinical results after use of the dual-component intramedullary implant [4], [5], [6]. However, Roukis and Coillard et al. found that mallet toe occurred in 2%–23% of cases after intramedullary PIP arthrodesis for hammer toe [7], [8]. Moreover, Lehman et al. [9] observed mallet toe in up to 40% of cases following PIP fusion performed for hammer toe, and Payo-Ollero et al. found mallet toe deformity in 70% of their cases [10].

Here, we report a case of mallet toe with penetration of the dorsal border of the middle phalanx head by the distal portion of a dual-component intramedullary implant following PIP arthrodesis for hammer toe that was successfully treated by removal of the implant and flexor tenotomy.

This work has been reported in line with the SCARE criteria [11].

## 2 Presentation of case

The patient provided written informed consent for publication of this case report.

**Fig. 1:**
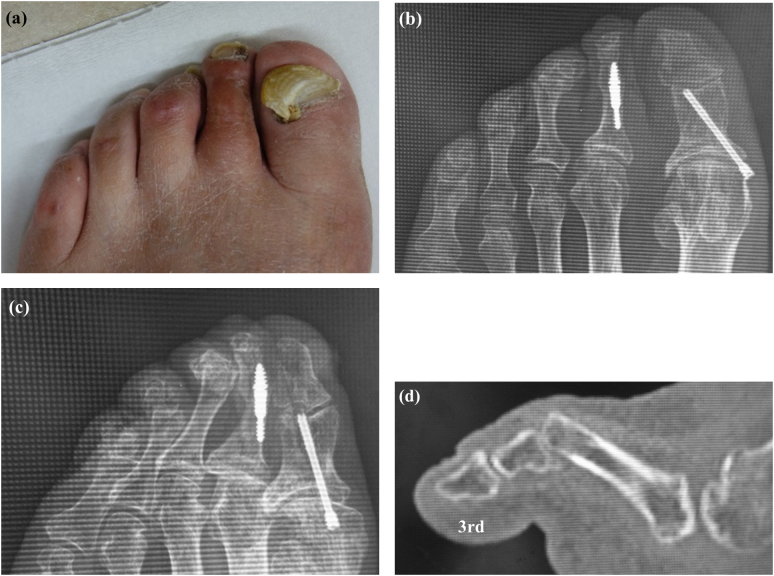
Clinical photograph showing callus formation over the third and fourth proximal interphalangeal joints (a). Radiographs showing third and fourth hammer toe deformity on anteroposterior (b) and oblique (c) views. Sagittal computed tomography image showing hyperflexion of the third proximal interphalangeal joint (d).

**Fig. 2:**
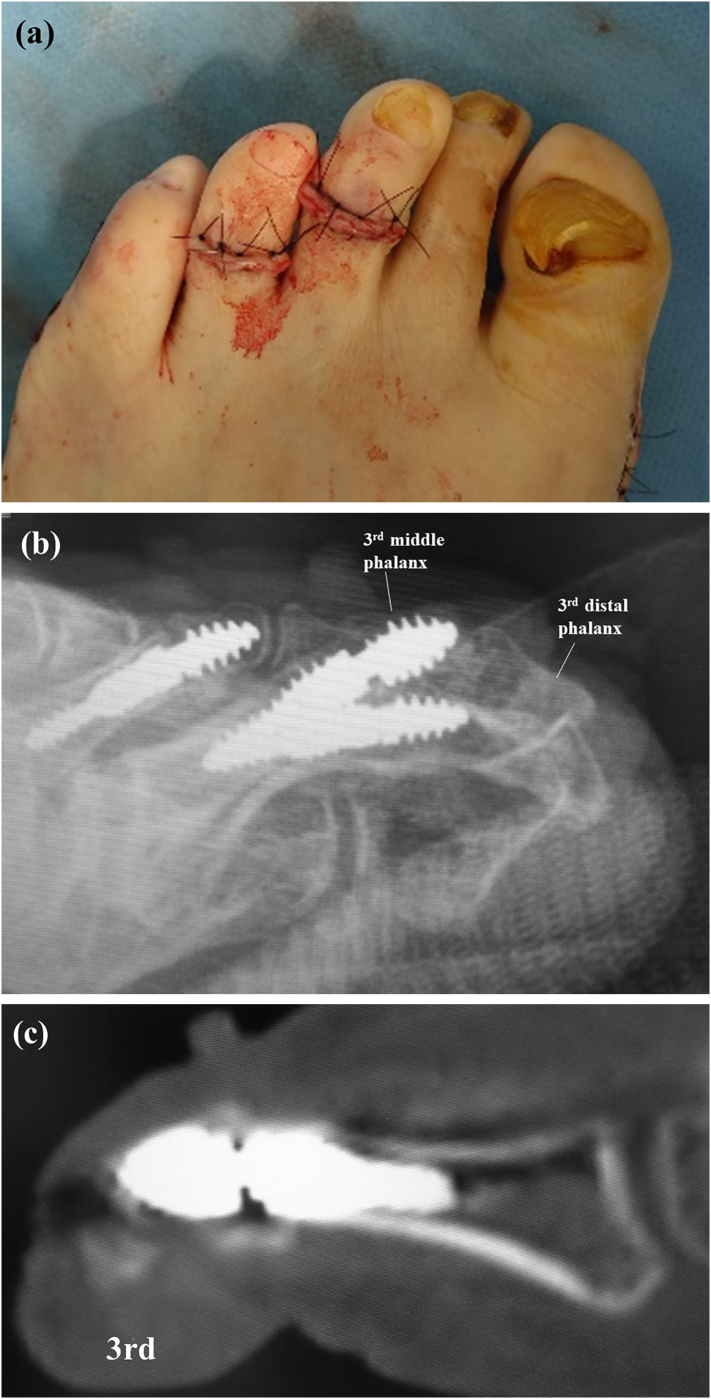
Intraoperative photograph showing correction of the hammer deformity of the third toe (a). A lateral radiograph (b) and sagittal computed tomography image (c) obtained soon after surgery confirm that the implant is correctly positioned.

At the time of presentation, the patient was 59 years old with a height of 177 cm, body weight of 76 kg, and body mass index of 24. He had left-sided incomplete hemiplegia as a result of a right-sided brain hemorrhage at the age of 52 years. At the age of 53 years, he was diagnosed as having diabetes. At the age of 54 years, he had undergone cervical laminoplasty for cervical ossification of the posterior longitudinal ligament, as well as placement of a coronary artery stent. At the age of 57 years, he had undergone arthrodesis of the second PIP using a dual-component intramedullary (Nextra) implant because of progressive second hammer toe deformity. At that time, we added the Akin osteotomy because the first toe pressed the second toe. Soon after the primary toe surgery, he reported pain when wearing shoes, formation of calluses and corns on the third and fourth PIPs, and a pain in the third toe (Fig. 1a). Radiographs obtained at that time showed hammer deformity of the third toe (Fig. 1b, c) and a sagittal computed tomography (CT) scan showed hyperflexion of the third PIP joint (Fig. 1d). Therefore, the third and fourth PIPs were arthrodesed using the dual-component intramedullary implant (Fig. 2a). Radiographs and a sagittal CT scan obtained soon after surgery confirmed that the implant was in the correct position (Fig. 2b, c).

**Fig. 3:**
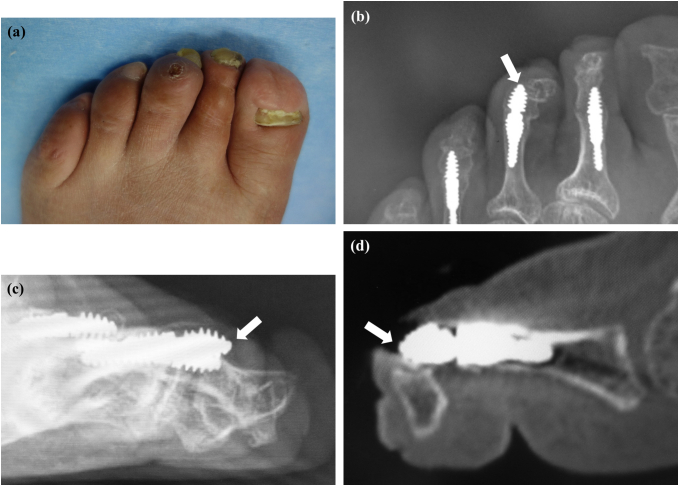
Clinical photograph showing swelling and ulceration over the dorsal surface of the distal interphalangeal joint in the third toe (a). Arrow showing the dorsal edge of the distal portion of the implant penetrating the dorsum of the middle phalanx head in a radiographic oblique view (b) and in lateral (c) and sagittal computed tomography scans (d).

Six months after arthrodesis of the third and fourth PIPs, the patient complained of pain and swelling in the left third toe. On physical examination, there was swelling, ulceration, and tenderness over the dorsum of the third distal interphalangeal (DIP) joint (Fig. 3a) with limited range of motion (−110/110). There was no sensory disturbance to the third toe. Radiographic and CT images showed that the distal portion of the implant had penetrated the thin dorsal cortex of the middle phalanx head and that there was hyperflexion of the DIP despite bony union of the PIP joint (Fig. 3b, c, d). We attributed these findings to development of mallet toe after PIP arthrodesis and made a plan to remove the implant and perform flexor tenotomy to correct the mallet toe. Before surgery, the Japanese Society for Surgery of the Foot (JSSF) lesser toe score was 15/100 (pain 0/40, function 15/45, alignment 0/15).

**Fig. 4:**
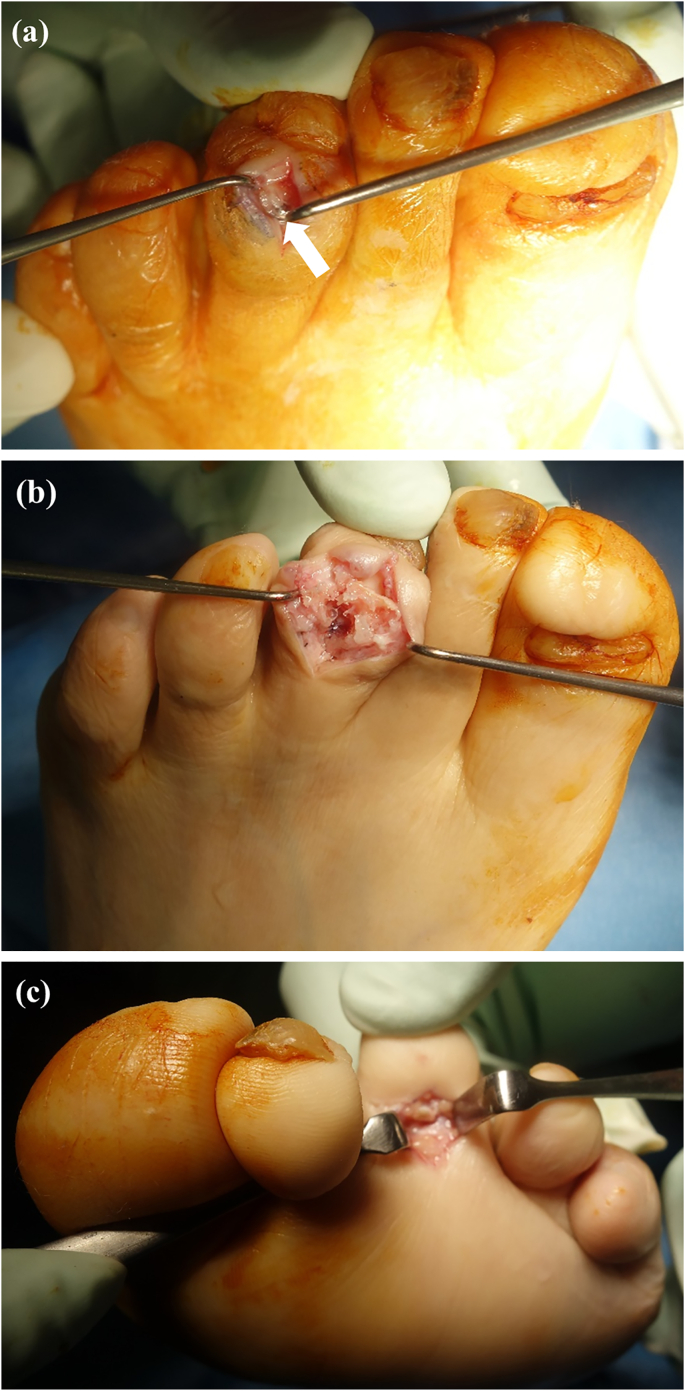
Intraoperative photographs. The arrow in (a) indicates the edge of the distal implant exposed on the dorsal surface of the middle phalanx head. The dorsal cortex over the middle and proximal phalanges was cut longitudinally and the implant was removed (b). Flexor tenotomy was performed via a short plantar incision (c).

**Fig. 5:**
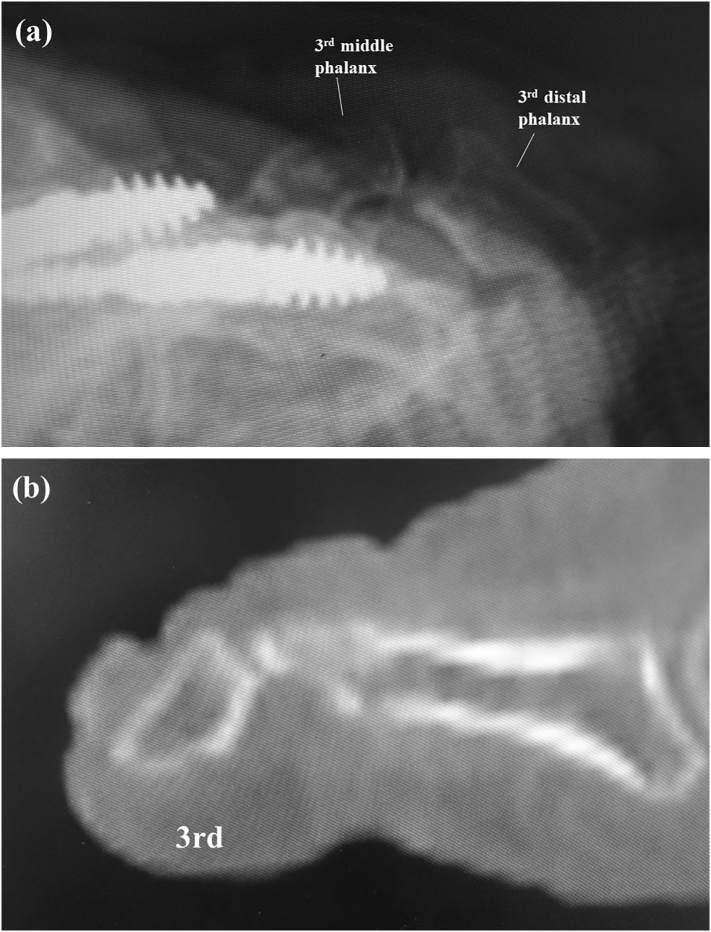
A lateral radiograph (a) and a sagittal computed tomography scan (b) show decreased hyperflexion of the distal interphalangeal joint.

**Fig. 6:**
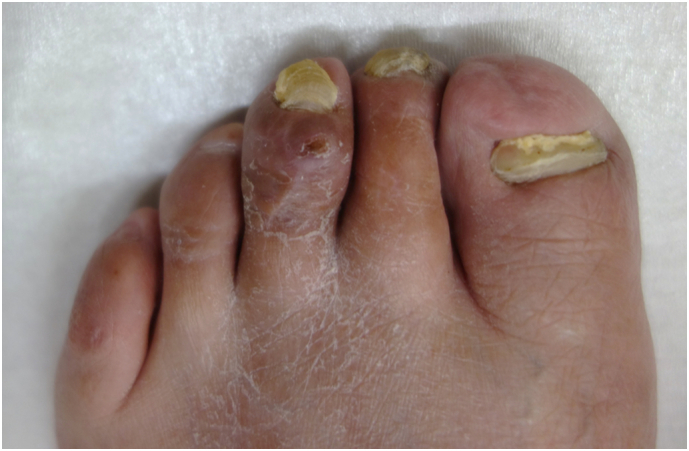
Mallet toe was corrected and swelling of the third toe was markedly decreased.

The surgery was performed by I.T. who graduated from the medical university in 2004 and was a foot and ankle surgeon. A 2-cm longitudinal incision was made over the dorsum of the middle and proximal phalanges of the third toe. The extensor tendon was split. Bony union of the PIP joint was observed but part of the distal portion of the implant was exposed at the dorsal aspect of the middle phalanx head (Fig. 4a). We pulled the exposed distal edge of the distal implant, but we could not remove the implant. Therefore, we made a longitudinal 3-mm incision in the dorsal cortex of the middle and proximal phalanges and removed the distal and proximal portions of the implant together (Fig. 4b). A short incision was made on the plantar aspect of the MTP joint and the flexor tendon was cut (Fig. 4c). The mallet toe was corrected and the hyperflexion of the DIP was adequately reduced. All skin incisions were closed with simple stitches. Mobilization and weightbearing were allowed as tolerated after surgery. Lateral radiographs and sagittal CT images obtained soon after surgery confirmed decreased hyperflexion of the DIP (Fig. 5a, b). The sutures were removed 2 weeks after surgery. By 3 weeks postoperatively, the patient was able to walk with full weightbearing on the forefoot and the third toe was straight with a marked reduction in swelling (Fig. 6).

At the 1-year follow-up visit, the patient was pain-free in daily activities and has had no recurrence of the lesser toe deformity. His JSSF lesser toe score has improved to 87/100 (pain 30/40, function 42/45, alignment 15/15).

## 3 Discussion

We have successfully treated a case of mallet toe with penetration of the dorsum of the middle phalanx head by the distal portion of a dual-component intramedullary implant that had been placed for PIP arthrodesis, by removal of the implant and flexor tenotomy. Jay et al. reported that they encountered difficulty when removing a two-piece intramedullary implant from a PIP joint fusion site [6]. Although easier to place, we encountered similar difficulty when attempting to remove this implant and needed to cut the dorsal cortex of the middle and proximal phalanges to do so.

One of the one-piece intramedullary implants that have been used for PIP arthrodesis is the Smart-Toe® monoblock device (Stryker, Mahwah, NJ, USA). The complications reported for this device include implant rupture (in 20.7% [12], 10.6% [13], 8% [10], 3% [14], and 2.4% [15] of cases) and migration (in 1.5% [14]). In a review of outcomes in toes treated with the StayFuse™ two-piece implant (Tornier; Wright Medical Group), Ellington et al. [5] identified bending of the implant in 5.2% of cases, implant breakage in 2.6%, and intraoperative fracture in 2.6%. Fazal et al. encountered implant-related complications in 5.3% of their cases, with breach of the cortex of the proximal phalanx in 0.6% and dissociation of the components in 4% [4]. However, there has been no report of penetration of the dorsal aspect of the middle phalanx head by the distal component of an appropriately placed dual-component intramedullary implant for correction of mallet toe so soon after PIP arthrodesis.

Payo Ollero et al. speculated that mallet toe might occur because the initial deformity causes retraction of the flexor tendons in the lesser toes which, after correction of the deformity in the PIP joint, causes stretching of the flexor tendons that produces a persistent flexion deformity of the DIP joint [10]. In our patient, we believe that the distal dorsal component of the implant may have penetrated the thin dorsal cortex of the middle phalanx head when the mallet toe progressed because he had diabetes and a history of incomplete hemiplegia due to cerebral infarction. Payo Ollero et al. recommended an additional surgical procedure such as flexor tenotomy or capsule release at the time of PIP arthrodesis or during follow-up to avoid mallet toe in the future [10]. Potential complications of tenotomy include recurrence of ulceration, postoperative infection, and need for amputation of the toe; however, Gilheany et al. reported that the complication rate was typically low after flexor tenotomy to address digital deformities [16]. Furthermore, in a series reported by Schmitz et al., ulceration recurred in 10.9% of cases, infection and bleeding developed in 4.0% and 1.0%, respectively, a second intervention was required in 1.0%, and amputation of the digit was needed in 1.0% [17]. We recommend close postoperative monitoring for these complications, although none occurred in our patient.

This report has two main limitations. The first is the short follow-up duration. The second is that only the lumbricals and interossei continue to work efficiently after tenotomy, which results in a lack of toe grasping with less propulsion of the toe, particularly during brisk walking and/or sporting activities. Therefore, careful follow-up is necessary. Kirschner wire (K-wire) fixation of the PIP joint can be option of surgery for hammer toe. However, the use of K-wire is not free from complications, such as the inconvinence of the patient, pin-track infection, phalanx rotation, recurrence of deformity, K-wire migration, accidental K-wire removal, lack of compression, K-wire breakage. For these reasons, we selected two-piece intramedullary implant for PIP joint fusiond to reduce such complications with use of K-wires.

## 4 Conclusion

We have successfully treated a rare case of mallet toe with penetration of the dorsum of the middle phalanx head by the distal portion of a dual-component intramedullary implant following PIP arthrodesis, by flexor tenotomy and implant removal. Additional flexor tenotomy could be performed at the same time as PIP arthrodesis in a patient who is thought to be at increased risk of development of lesser toe deformity after surgery.

## Funding

This research received no specific grant from any funding agency in the public, commercial, or not-for-profit sectors.

## Ethical approval

A clinical case report is exempt from ethical approval in our institution.

## Consent

A written informed consent was obtained from the patient for publication of this case report and accompanying images. A copy of the written consent is available for review by the Editor-in-Chief of this journal on request.

## Authors' contributions

IT was responsible for this study, and managed this study. All authors read and approved the final manuscript.

## Registration of research studies

None.

## Provenance and peer review

Not commissioned, externally peer-reviewed.

## Guarantor

Ichiro Tonogai.

## Availability of data and materials

All data generated or analyzed during this study are included in this published article. No supplementary file is not included with this manuscript.

## Declaration of competing interest

The Authors declare that there is no conflict of interest.
